# Insights for applying N,S-doped carbon dots as a fluorescent nanoprobe for estimation of some nitro-calcium channel blockers

**DOI:** 10.1098/rsos.220609

**Published:** 2022-10-26

**Authors:** Shymaa M. Abd Elhaleem, Sh. Shalan, F. Belal, F. Elsebaei

**Affiliations:** Department of Pharmaceutical Analytical Chemistry, Faculty of Pharmacy, Mansoura University, Mansoura 35516, Egypt

**Keywords:** nitrogen/sulfur carbon quantum dots, nicardipine, nifedipine, nimodipine, inner filter effect

## Abstract

A facile and simple one-step hydrothermal approach was adopted for fabrication of N and S co-doped carbon quantum dots probe (NSCDs) by using thiosemicarbazide as a dopant and citric acid as a precursor. The prepared NSCDs with a high quantum yield of 0.58 were characterized using UV–visible spectroscopy, IR spectroscopy and high-resolution transmission electron microscopy. The as-obtained NSCDs could be deemed as an effective fluorescent nanosensor for the determination of some anti-hypertensive nitro-calcium channel blockers (Nitro-CCBs) including nicardipine (NIC), nifedipine (NIF) and nimodipine (NIM) whether in pure form or in their pharmaceutical formulations. Measurements of NSCD emission intensity were performed at 416 nm after being excited at 345 nm. Nitro-CCBs could induce quenching in the native fluorescence of NSCDs due to the inner filter effect and static quenching mechanism. The studied compounds were investigated within linear detection range of (10.0–100.0 µM) for NIC, (5.0–60.0 µM) for NIF and (5.0–60.0 µM) for NIM. Correlation coefficients are greater than or equal to 0.9998 and detection limits are ranged between 0.55 and 1.86 µM. The proposed method was extended to estimate the studied compounds in different pharmaceutical samples with high % recoveries ranging from (97.95 to 101.28%) and low % relative standard deviation values (less than 2%). Validation of the developed spectrofluorimetric method was done along with the International Council of Harmonization requirements.

## 1. Introduction

Calcium channel blockers (CCBs) are mostly and extensively used for the treatment of several indications including angina, hypertension, coronary heart disease and cardiac dysrhythmias. CCBs are a heterogeneous group of compounds with a specific structure and consequently distinctive pharmacological influences. They act through inhibition and blocking of calcium ion influx via calcium channels located in the cell membrane. CCBs are classified into three subclasses including phenylalkylamines, benzodiazepines and dihydropyridines, exemplified by nicardipine, nifedipine and nimodipine [1].

**Figure 1.**
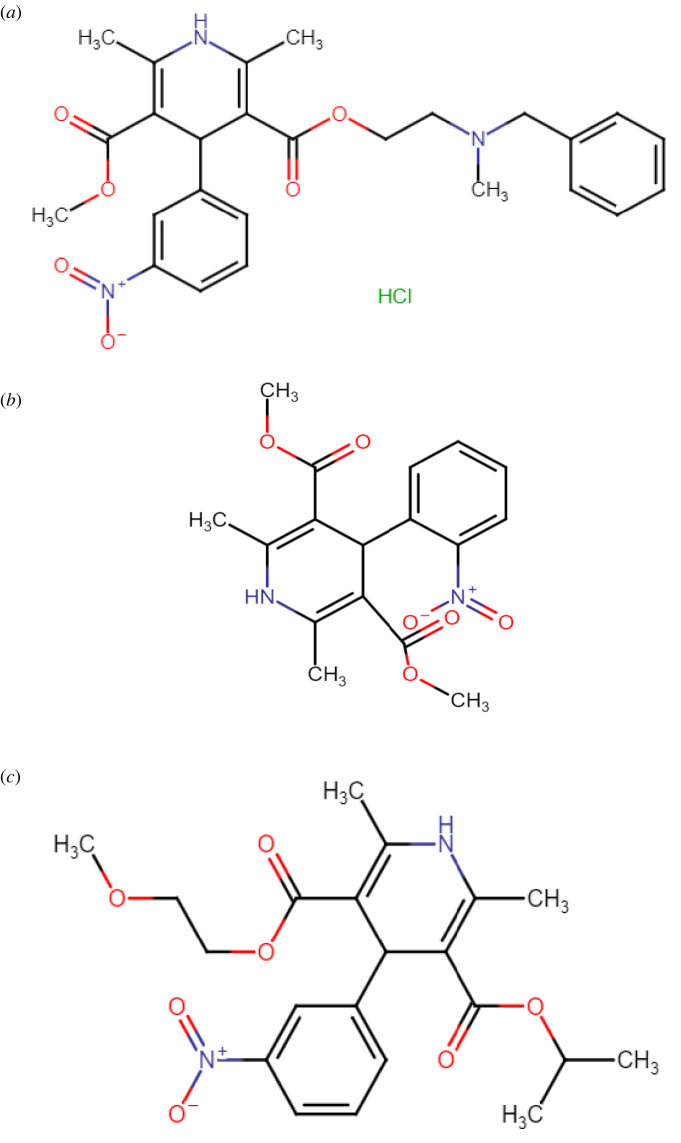
Structural formulae of nicardipine hydrochloride (*a*), nifedipine (*b*) and nimodipine (*c*).

The compounds studied were nicardipine hydrochloride [NIC] (figure 1*a*) 2-[benzyl(methyl)amino]ethylmethyl-1,4-dihydro-2,6-dimethyl-4-(3-nitrophenyl)pyridine-3,5-dicarboxylate hydrochloride [2], nifedipine [NIF] (figure 1*b*) dimethyl-1,4-dihydro-2,6-dimethyl-4-(2-nitrophenyl)pyridine-3,5-dicarboxylate and nimodipine [NIM] (figure 1*c*) 3-*O*-(2-methoxyethyl)-5-*O*-propan-2-yl-2,6-dimethyl-4-(3-nitrophenyl)-1,4-dihydropyridine-3,5-dicarboxylate [3]. Several reports are found in the literature for the three compounds in their pharmaceutical dosage forms and biological fluids; for NIC: spectrophotometric [4–8], spectrofluorimetric (SF) [9,10], high-performance liquid chromatography (HPLC) [11–13], gas chromatography (GC) [14] and electrochemical [15] methods were reported. For NIF: spectrophotometric [5,16,17], SF [10], HPLC [18,19] and electrochemical methods [20,21] were also described. Similarly, for NIM: SF [22,23], spectrophotometric [24,25], HPLC [26,27] and electrochemical methods [28,29] were found in the literature.

The reported spectrofluorimetric methods for NIC [9,10], NIF [10] and NIM [22,23] are tedious and need prior chemical derivatization, while the proposed study is a green, simple, direct and the first spectrofluorimetric method for estimation of the studied compounds without derivatization. This study is based on the quenching of N and S co-doped carbon quantum dot (NSCD) fluorescence after addition of increasing concentrations of NIC, NIF and NIM.

Recently, fluorescent carbon quantum dots (FCQDs) are generally defined as newly discovered fascinating members of carbon-based nanomaterials. They represent the main point of interest of many researchers because of their remarkable and distinguishable key features including small size (typically around 10 nm), tunable photoluminescence (PL), eco-friendliness, negligible cytotoxicity, superior biocompatibility, high-temperature stability, robust chemical inertness and easy routes for their preparation [30–33]. Different types of FCQDs with different surface functional groups have been prepared depending on the method and precursors used during their synthesis [34]. FCQDs have different functional groups like carboxyl, carbonyl and hydroxyl resulting in their water solubility. FCQDs are considered as superior alternatives and promising replacements to traditional semiconductor quantum dots (QDs) to overcome difficulties including complicated synthesis procedures and toxicity resulting from their metal content (e.g. Cd, Zn, Se) [35–37]. Introduction of heteroatoms including nitrogen, sulfur, phosphorus and other heteroatoms occurs to improve the fluorescence intensity of carbon dots. Literature demonstrates that doping of N, P and S atoms can efficiently increase the quantum yield (QY), reduce the energy gap and make the luminescence redshift [38–40]. Mainly, the surface structure of the prepared NSCDs is carbon source doping with N and S heteroatoms and consequently emerging their inherent fluorescence. In this work, NSCDs were easily prepared by using available starting materials in a cost-effective manner (one-pot hydrothermal method) which adds an advantage to the study over other reported NSCD synthesis methods including chemical oxidation methods [41], microwave-assisted methods [42] and carbonizing organics methods [43].

The inner filter effect (IFE) is considered as a source of errors in fluorimetry indicating the absorption of the excitation and/or emission of light by absorbers (quenchers) in the detection system, but recently it is exploited in new fluorescence assessments. IFE is based on converting absorption signals into fluorescence signals which may occur when the absorption spectrum of interfering absorbers possesses a complementary overlap with the fluorescence excitation or emission spectra of fluorophores [44,45].

Herein, we report a simple and low-cost procedure to synthesize NSCDs from citric acid (CA) through one-step thiosemicarbazide (TSC) assisted reflux treatment. In addition, NSCDs could be applied as a selective fluorescent sensing platform for the estimation of some Nitro-CCBs including NIC, NIF and NIM. Different factors affecting the fluorescence intensities were studied, including pH of the medium and incubation time. After optimization of the final experimental parameters, NSCDs were validated as a fluorescent probe for promising detection of the studied drugs and subsequently applied to pharmaceutical formulations. Moreover, possible mechanisms of fluorescence quenching of NSCDs were investigated through IFE and Stern–Volmer equation.

## 2. Experimental set-up

### 2.1. Materials and reagents

Pure nicardipine hydrochloride, nifedipine and nimodipine with labelled purities of 99.60%, 99.82% and 99.20% were kindly donated by global NAPI, EPICO and Pharco Pharmaceutical Co., Egypt, respectively. Epilat retard^®^ Tablets; batch no. 1911216 (product of EPICO Pharmaceutical Co., 10th of Ramadan City, Egypt), labelled to contain 20 mg NIF/tablet. Adalat LA^®^ Tablets (batch no. BXHNNK), labelled to contain 30 mg NIF/tablet, manufactured by Bayer Pharma AG Pharmaceutical Co., Jeddah, Saudi Arabia. Nimodipine^®^ Tablets; batch no. 102 (product of Pharco Pharmaceutical Co., Egypt), labelled to contain 30 mg NIM/tablet. All pharmaceutical formulations were purchased from a local pharmacy in the Egyptian market.

Sodium acetate, acetic acid 96%, boric acid, sodium hydroxide, ethanol, CA and TSC were obtained from Sigma-Aldrich (USA). Ultra-pure water was used throughout the study; besides, all chemicals were of analytical reagent grade.

### 2.2. Instrumentation and characterization

The fluorescence spectra were recorded using a Cary Eclipse fluorescence spectrophotometer at an excitation wavelength of 345 nm. Ultraviolet–visible (UV–Vis) absorption spectra were recorded with a 1601 spectrophotometer (Shimadzu) with 1 cm quartz cell. The IR spectra were recorded using an IS10-Nicolet Fourier transform infrared (FT-IR) spectrophotometer (USA) in the range of 4000–400 cm^−1^ and operated at 8 cm^−1^ resolution as 32 scans. The particle size distributions of NSCDs were observed on a high-resolution transmission electron microscope (HRTEM), model JSM-2100 (JEOL, Japan) operating at an accelerating voltage of 200 kV. All measurements were conducted in triplicate at room temperature.

### 2.3. Synthesis of NSCDs

In this work, an eco-friendly NSCDs were directly fabricated from TSC and CA via a one-pot hydrothermal process. Briefly, 0.52 g of citric acid powder and 0.68 g of thiosemicarbazide were dissolved in 20 ml of ultra-pure water. The reaction was held at 180°C for 8 h for converting starting colourless solution, then pale yellow to finally orange, fluorescent solution of NSCDs (1.0 g l^−1^). The resulting solution was purified via filtration to remove large particles before being stored in a refrigerator for further use [46].

### 2.4. Standard stock solutions and buffer solutions

In order to obtain stock solutions of NIC, NIF and NIM (1.0 mM), accurately weighed equivalent amounts of the powder of the studied drugs were dissolved in 10 ml of ethanol in 100 ml measuring flasks, followed by sonication for 5 min for complete dissolution, then completed to the mark with the same solvent. The working solutions with different concentrations were obtained after appropriate dilution of the stock solution with ultra-pure water. All drug solutions were light-protected and stored in a refrigerator to avoid photo-degradation.

The ultimate working solution of NSCDs was obtained after transferring 5 ml of the stock solution (1.0 g l^−1^) into a 50 ml measuring flask then diluted to the mark with ultra-pure water.

Acetate and borate buffers (0.2 M) were prepared according to the United States Pharmacopeia (USP) [47] with pH ranges of (3.7–5.7) and (6.5–12), respectively, throughout the study.

### 2.5. Quantum yield measurement

The QY of the NSCDs was measured using quinine sulfate (QS) as the standard, comparing the integrated fluorescence intensity and the absorbance of the NSCD suspensions with those of the standard. The QS was dissolved in 0.1 M sulfuric acid (QY: 0.54 at 350 nm). The QY of the dots was determined through an established procedure according to the following equation [48]:

2.1 $$\phi_{NS\mathrm{CDs}}=\phi_{\mathrm{QS}}\times \left[\frac{F_{NS\mathrm{CDs}}}{F_{\mathrm{QS}}}\right]\times \left[\frac{A_{\mathrm{QS}}}{A_{NS\mathrm{CDs}}}\right]\times {\left[\frac{\eta_{NS\mathrm{CDs}}}{\eta_{\mathrm{QS}}}\right]}^{2},$$

where *ϕ* represents the QY, ***F*** refers to the measured integrated fluorescence emission intensity, ***A*** is the absorbance, and ***η*** refers to the solvent refractive index. In aqueous solutions *η****_N_***_***S***_**_CDs_**/*η***_QS_** is equal to 1.

### 2.6. Procedures

#### 2.6.1. Calibration graph construction

In this work, graphs of studied drugs were obtained after the addition of 0.1 ml of NSCDs to different volumes from standard solutions of each drug in a 10 ml calibrated flask. The volume was completed to the mark with ultra-pure water and mixed well to obtain solutions in the range of (10.0–100.0 µM), (5.0–60.0 µM) and (5.0–60.0 µM) for NIC, NIF and NIM, respectively. Fluorescence was recorded at emission peak of around 416 nm (*λ*_ex_ = 345 nm) against an appropriate reagent blank prepared simultaneously for precise determination of the relative fluorescence intensity (RFI). Then, the corresponding regression equations were concluded from the data relating RFI and concentrations of each drug (in µM).

#### 2.6.2. Analysis of drugs in their pharmaceutical formulations

Ten pulverized tablets of Epilat retard^®^, Adalat LA^®^ and Nimodipine^®^ were separately mixed. Subsequently, transferring of precisely weighed quantities of the powder equivalent to (1.0 mM) for each of NIF and NIM into 100 ml calibrated flasks and addition of 50 ml of ethanol were done. Then sonication for 30 min was performed to ensure complete dissolution of the drugs. Upon filtration into a 100 ml measuring flask, ethanol was added to the mark. Serial dilutions with ultra-pure water were needed to get solutions within the working range of the method, and thereafter, the procedure described in §2.6.1 was applied. The nominal contents of the tablets were estimated adopting the corresponding regression equations.

## 3. Results and discussion

### 3.1. Optical characteristics of NSCDs

**Figure 2.**
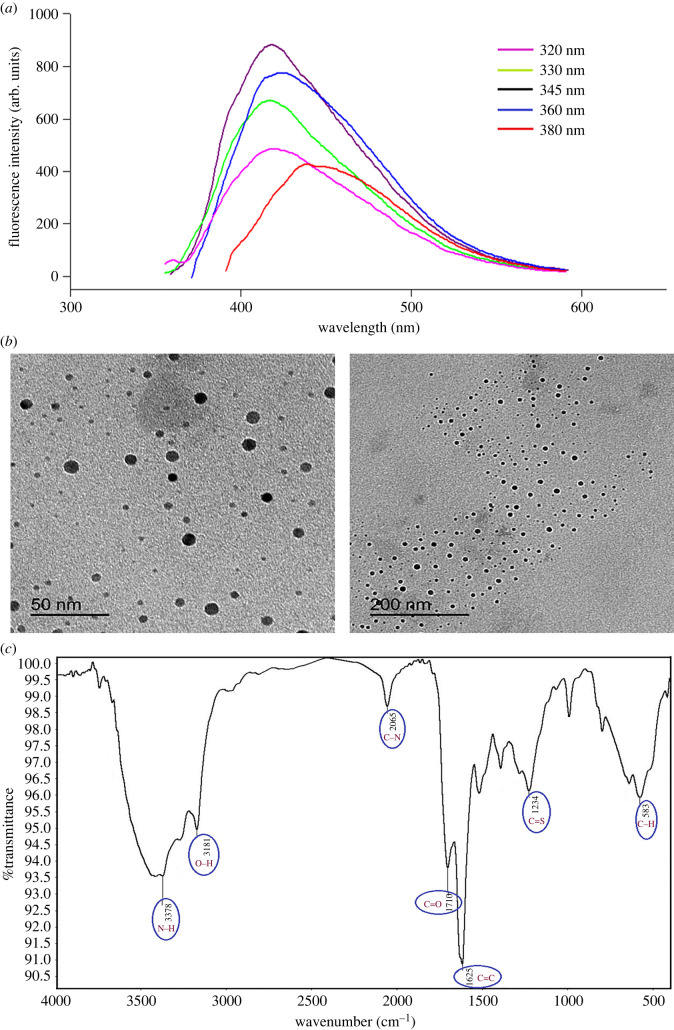
(*a*) Fluorescence spectra of the NSCDs at different excitation wavelengths. (*b*) Typical HRTEM images of the NSCDs at two different magnifications. (*c*) FT-IR spectrum of NSCDs.

UV spectroscopy is a straightforward and useful tool used to study the structural changes of TSC, CA and NSCDs (electronic supplementary material, figure S1). It was observed that NSCDs showed an obvious UV–Vis absorption peak at about 310 nm, which is attributed to the trapping of excited-state energy by the surface states, leading to strong emission [49]. It was also found that NSCDs exhibit an intense fluorescence emission at 416 nm after getting excited at 345 nm owing to emissive traps of nitrogen and sulfur atoms (electronic supplementary material, figure S2). The emission wavelength of NSCDs is excitation-dependent, so the best emission fluorescence was set and chosen after changing the excitation wavelength over the range 320–380 nm (figure 2*a*).

As evident from HRTEM images of the prepared NSCDs, it could be clearly observed that NSCDs are distributed as spherical particles with uniform sizes ranging from 10 to 13 nm (11.5 nm average diameter in size; figure 2*b*).

For further confirmation, figure 2*c* provides the FT-IR spectrum of NSCDs with some main absorption peaks. The broad peaks around 3378 and 3181 cm^−1^ correspond to the stretching vibrations of N–H and O–H groups which impart ideal hydrophilicity to the product. Additionally, the peaks centred at 2065, 1710, 1625, 1234, 579 cm^−1^ were attributed to C–N, C=O, C=C, C=S and C–H groups, respectively [50]. As can be seen, FT-IR analysis was in good agreement with HRTEM images and UV–Vis spectroscopy measurements which altogether prove successful synthesis of NSCDs.

### 3.2. Response mechanism

**Figure 3.**
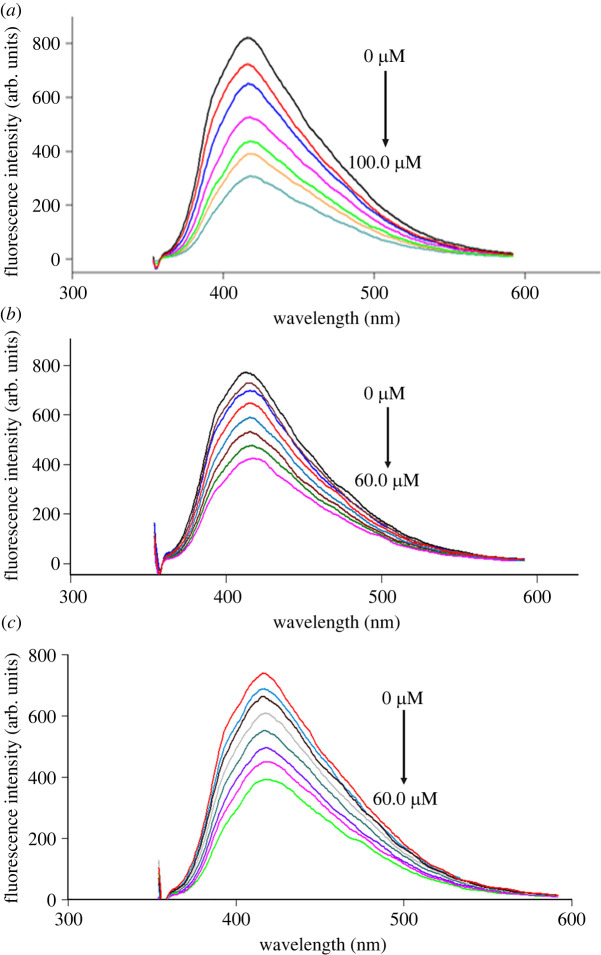
Fluorescence emission spectra of the NSCDs in aqueous solution upon addition of various concentrations of NIC (*a*) from top to bottom: 0 µM, 10.0 µM, 30.0 µM, 50.0 µM, 70.0 µM, 80.0 µM, 100.0 µM; NIF(*b*) from top to bottom: 0 µM, 5.0 µM, 10.0 µM, 20.0 µM, 30.0 µM, 40.0 µM,50.0 µM, 60.0 µM; NIM (*c*) from top to bottom: 0 µM, 5.0 µM, 10.0 µM, 20.0 µM, 30.0 µM, 40.0 µM,50.0 µM, 60.0 µM.

**Scheme 1.**
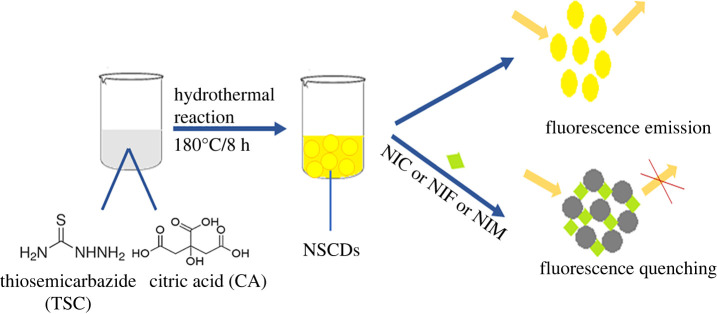
General procedure for synthesis and application of NSCDs.

At high temperatures, the reaction between CA and TSC leads to the generation of dots abundant with hydroxyl, carboxyl, amino and sulfur groups. Upon the intimate contact between NSCDs and Nitro-CCBs, NSCDs lose their strong native fluorescence (*λ*_ex_ 345 nm/*λ*_em_ 416 nm). The outline of the mechanism of the developed method is presented in scheme 1. As indicated in figure 3, the fluorescence intensity of NSCDs at 416 nm decreased dramatically with increasing concentration of NIC, NIF and NIM. In this manner, different concentrations of the drugs were added to ‘turn off' the fluorescence of NSCDs and their fluorescence spectra were recorded.

**Figure 4.**
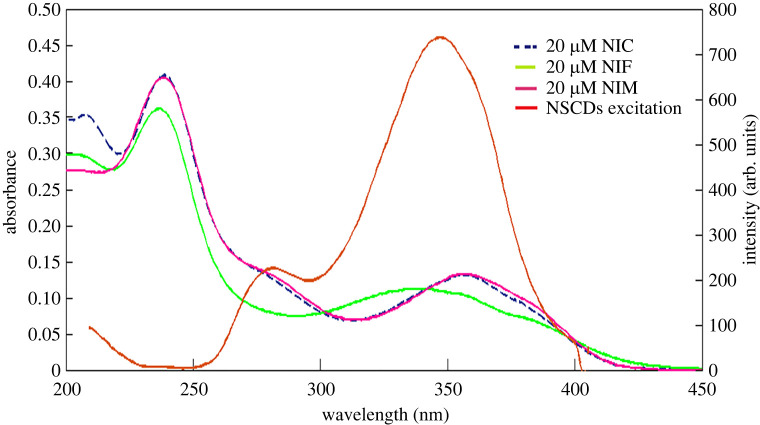
A co-plot showing the the great overlap between UV–Vis absorption spectra of NIC, NIF and NIM and fluorescence excitation of the NSCDs.

The sensing mechanisms of fluorescence quenching mainly involve static quenching, dynamic quenching, IFE and fluorescence resonance energy transfer (FRET). IFE might occur because of overlapping between UV absorption bands of Nitro-CCBs and NSCD excitation spectrum (figure 4). For possible IFE, it was necessary to correct the fluorescence intensity of NSCDs upon addition of increasing concentrations of the quenchers according to the following equation:

3.1 $$F_{\mathrm{corr}}=F_{\mathrm{obs}}\times \;10^{A_{\mathrm{ex}}+A_{\mathrm{em}}/2},$$

where *F*_obs_ refers to the observed fluorescence intensity, *F*_corr_ refers to the corrected fluorescence intensity upon removing IFE from *F*_obs_ and *A*_ex_ and *A*_em_ refer to the absorbance of the quencher at the excitation and emission wavelength of the fluorophore (NSCDs), respectively.

Consequently, determination of the suppressed efficiency (%*E*) was applied for observed and corrected fluorescence intensity along with the following equation:

3.2 $$\text{\%}E=\left[1-\left(\frac{F}{F^{\circ }}\right)\right]\times 100.$$

**Figure 5.**
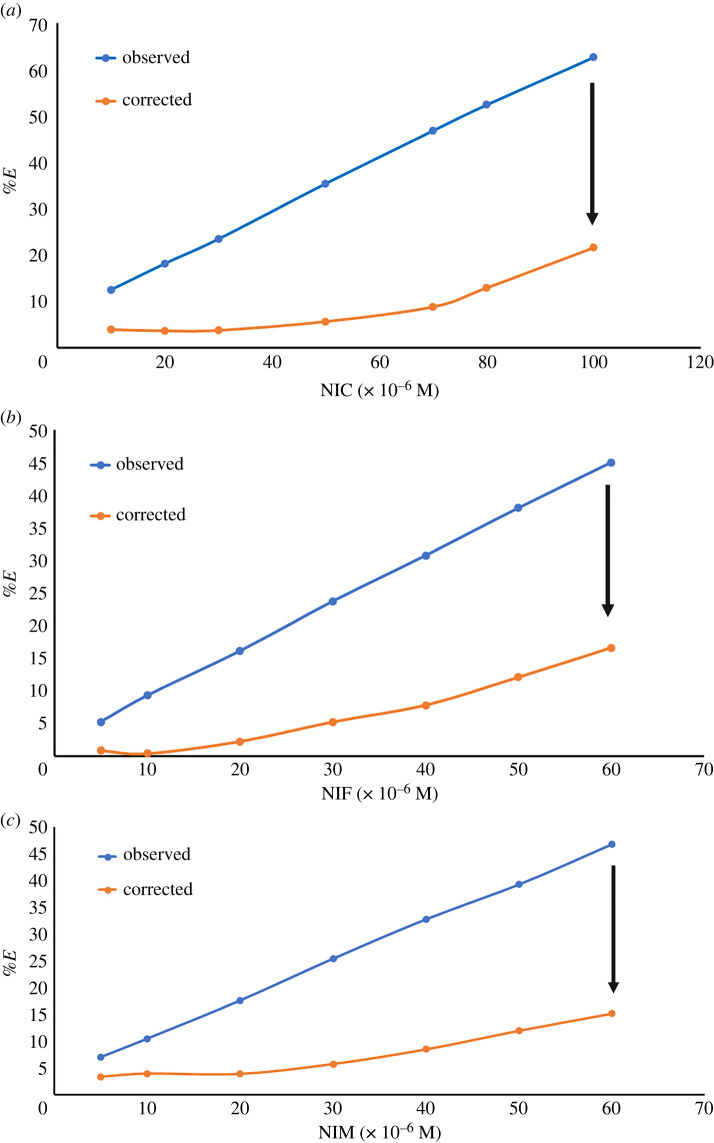
Suppressed efficiency of observed and corrected fluorescence of NSCDs after addition of different concentrations of NIC (*a*), NIF (*b*) and NIM (*c*).

The plots between %*E* of both observed and corrected fluorescence intensity of NSCDs and concentration of drugs (in M) indicate that one of the quenching mechanisms throughout this study was IFE (figure 5) [51].

To illustrate the possible mechanism that might be responsible for the remaining part of NSCD quenching, the Stern–Volmer equation was adopted [37]:

3.3 $$\frac{\;\;F^{0}}{F}=1+K_{\mathrm{sv}}[Q],$$

where *F*^0^ and *F* refer to fluorescence emission intensities in case of absence and presence of the quencher, respectively; *K*_sv_ represents the Stern–Volmer quenching constant; [*Q*] represents the concentration of the quencher.

**Figure 6.**
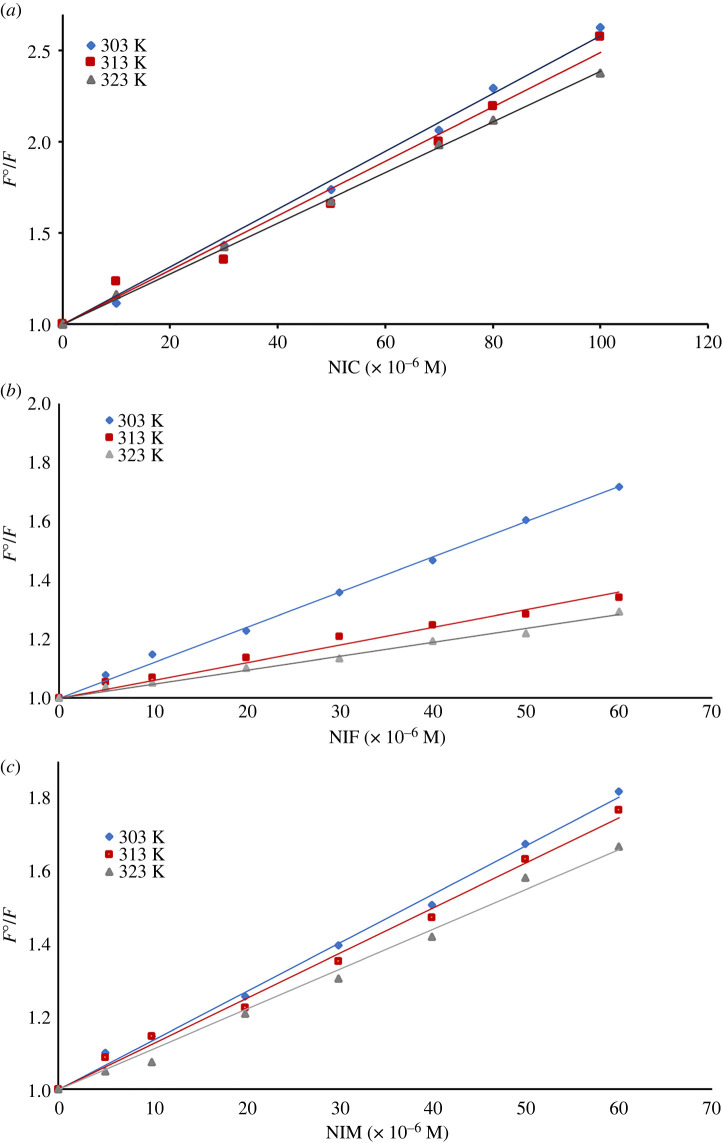
Stern–Volmer plots between *F*^0^/*F* and concentration of NIC (*a*), NIF (*b*) and NIM (*c*).

**Scheme 2.**
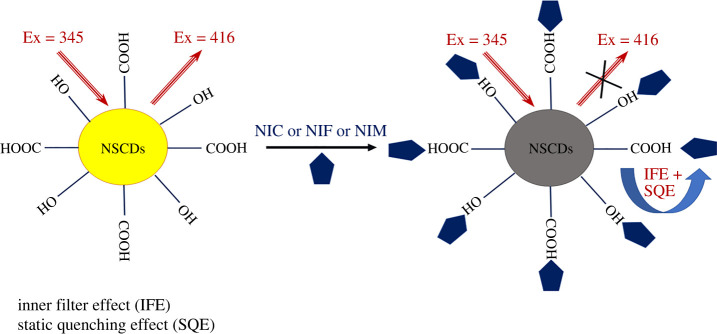
Schematic illustration of the quenching mechanisms of NSCDs with NIC, NIF and NIM.

Additionally, Stern–Volmer plots for each drug were studied at three different temperatures (303, 313 and 323 K) as illustrated in figure 6. It was observed that, as the temperature increases, the values of *K*_sv_ decrease consequently, confirming the possibility of static quenching process [52]. Based on these findings, the quenching of the inherent fluorescence of the prepared dots was due mainly to both IFE and static quenching mechanisms, as illustrated in scheme 2.

### 3.3. Method optimization

#### 3.3.1. Influence of buffer pH

To study the influence of pH on the analytical response, acetate and borate buffers were used at different pH levels ranging from 3.7 to 12. Based on the obtained results, it was found that an increase in pH values ​​above 8 led to a decrease in the solubility of drugs. On the other hand, it was also noticed that changing pH values over the range 3.7–12 resulted in no variation in the measurements. As a result, the proposed approach was conducted without the use of buffers.

#### 3.3.2. Impact of incubation time

Incubation time is an essential factor that is needed to study the interaction between NSCDs and the three drugs. Incubation times ranging from 1 to 25 min were attempted. From the obtained results, it was deduced that the completion of the reaction was done in less than a minute and remained stable for more than 25 min. Consequently, the approach was performed immediately after mixing the analyte solution with the sensing material.

### 3.4. Validation of the developed method

Investigation of method validation was performed according to ICH Q2R1 recommendations [53].

The fluorescence of prepared dots was recorded at emission peak of around 416 nm before and after addition of increasing concentrations of the studied drugs. The linearity of the proposed method was checked between the decrease in the fluorescence intensity of NSCDs and the respective drug concentrations in the range of (10.0–100.0 µM), (5.0–60.0 µM) and (5.0–60.0 µM) for NIC, NIF and NIM, respectively. All the data showing the linearity of the developed method with good correlation coefficient are documented in table 1.

**Table 1.** Analytical performance data for the determination of NIC, NIF and NIM by the proposed method.

parameter	value		
	NIC	NIF	NIM
wavelength (nm)	345/416 nm		
linearity range	10.0–100.0 (µM)	5.0–60.0 (µM)	5.0–60.0 (µM)
intercept (*a*)	57.146	16.051	24.961
slope (*b*)	4.619	5.511	5.359
correlation coefficient (*r*)	0.9998	0.9999	0.9998
s.d. of residuals (*S_y/x_*)	3.540	1.275	2.266
s.d. of intercept (*S_a_*)	2.598	0.917	1.629
s.d. of slope (*S_b_*)	0.043	0.025	0.045
percentage relative standard deviation, % RSD	0.97	0.95	1.12
percentage relative error, % error	0.37	0.36	0.42
LOD	1.86 (µM)	0.55 (µM)	1.00 (µM)
LOQ	5.62 (µM)	1.66 (µM)	3.00 (µM)

Mathematical calculations of quantification limit (LOQ) and limit of detection (LOD) were performed adopting ICH Q2R1 recommendations [53]. The calculated values of LOQ and LOD for each drug are presented in table 1, pointing out that the method has sufficient sensitivity. LOQ = 10*S_a_*/*b* and LOD = 3.3*S_a_*/*b,* where *S_a_* = standard deviation of the intercept of the calibration curve and *b* = slope of the calibration curve.

For evaluation of the accuracy of the developed approach, it was essential to compare the obtained results for the three drugs with those found using comparison methods [10,23]; both for the raw materials and the commercial dosage forms. Based on statistical analysis of the data obtained by both methods, regarding both Student's *t*-test and variance ratio *F*-tests [54], it was deduced that there is no significant variance concerning the accuracy and precision between the methods [10,23] as shown in table 2.

**Table 2.** Application of the proposed method for the determination of NIC, NIF and NIM in pure forms.

	NIC			NIF			NIM		
	amount taken (µM)	amount found (µM)	percentage found^a^	amount taken (µM)	amount found (µM)	percentage found^a^	amount taken (µM)	amount found (µM)	percentage found^a^
parameters	10.0	9.97	99.70	5.0	5.06	101.20	5.0	5.02	100.40
	20.0	19.93	99.65	10.0	10.05	100.50	10.0	9.88	98.80
	30.0	29.53	98.43	20.0	19.64	98.20	20.0	19.72	98.60
	50.0	50.42	100.84	30.0	30.19	100.63	30.0	30.37	101.23
	70.0	70.65	100.93	40.0	40.0	100.00	40.0	40.53	101.33
	80.0	80.57	100.71	50.0	50.16	100.32	50.0	49.59	99.18
	100.0	99.03	99.03	60.0	59.88	99.80	60.0	59.91	99.85
mean ($\bar{X}$)			99.90			100.09			99.91
±s.d.			0.97			0.95			1.12
%RSD			0.97			0.95			1.12
% error			0.37			0.36			0.42
*N*	7			7			7		
	comparison method [10]						comparison method [23]		
mean ± s.d.	99.97 ± 0.62			100.25 ± 1.75			99.81 ± 1.46		
*N*	3			3			4		
*t*-test	0.14 (2.306)^b^			0.15 (2.306)^b^			0.12 (2.262)^b^		
*F*-value	2.45 (19.33)^b^			3.39 (5.14)^b^			1.70 (4.76)^b^		

^a^ Mean of three determinations.

^b^The values in parentheses are the tabulated *t-* and *F*-values at *p* = 0.05.

Screening of method precision was affirmed by detection of intra-day and inter-day precisions through determination of three replicates of three different concentrations of each drug. Intra-day precision was achieved within the same day, but inter-day precision was attained on three sequential days. As depicted in table 3, the small values of relative standard deviation (%RSD), less than 2%, indicate good reproducibility and precision.

**Table 3.** Intra-day and inter-day precision data^a^ for the determination of the studied drugs by the proposed method.

parameters		drug concentration (μM)								
		NIC			NIF			NIM		
		30.0	50.0	70.0	30.0	40.0	50.0	30.0	40.0	50.0
intra-day	mean	99.33	99.27	101.07	99.47	100.85	100.54	101.06	100.71	99.16
	± s.d.	±1.15	±1.71	± 1.32	± 1.60	± 0.68	± 0.51	± 0.89	± 0.67	± 0.54
	% RSD	1.16	1.72	1.31	2.01	0.67	0.51	0.88	0.67	0.54
	% error	0.67	0.99	0.76	1.16	0.39	0.29	0.51	0.39	0.31
inter-day	mean	99.74	101.09	100.78	99.48	99.82	99.82	99.75	99.61	99.27
	± s.d.	±1.61	±0.99	±1.61	± 0.57	± 1.67	± 1.43	± 1.30	± 0.49	± 1.05
	% RSD	1.61	0.98	1.60	0.57	1.67	1.43	1.30	0.49	1.06
	% error	0.93	0.57	0.92	0.33	0.96	0.83	0.75	0.28	0.61

^a^Each result is the average of three separate determinations.

### 3.5. Applications

#### 3.5.1. Analysis of NIF and NIM in pharmaceutical preparations

Based on the found percentage recoveries, the proposed method could be easily used to determine Nitro-CCBs in their dosage forms without any interference from common additives and excipients. The results of both the proposed and comparison methods [10,23] were subjected to statistical analysis. Therefore, no significant differences regarding accuracy and precision were presented because the calculated values were lower than the tabulated values for both Student's *t*-test and variance *F*-tests (table 4).

**Table 4.** Determination of NIF and NIM in pharmaceutical preparations using the proposed method.

parameters	proposed method			comparison methods [10,23]		
	amount taken (µM)	amount found (µM)	percentage found^a^	amount taken (µM ml^−1^)	amount found (µM ml^−1^)	percentage found^a^
Epilat retard^®^ tablet (20 mg NIF/1 tab)	30.0	29.91	99.71	1.0	1.01	101.00
	40.0	39.73	99.33	2.0	1.99	99.50
	50.0	50.02	100.04	4.0	4.01	100.25
mean			99.69			100.25
± s.d.			0.36			0.75
% RSD			0.36			0.75
% error			0.21			0.43
*t*-test	1.17 (2.776)^b^					
*F*-value	4.34 (19.00)^b^					
Adalat LA^®^ tablet (30 mg NIF/1 tab)	30.0	29.65	98.84	1.0	1.02	102.00
	40.0	39.43	98.58	2.0	1.97	98.50
	50.0	50.37	100.74	4.0	4.01	100.25
mean			99.39			100.25
± s.d.			1.18			1.75
% RSD			1.19			1.75
% error			0.69			1.01
*t*-test	0.70 (2.776)^b^					
*F*-value	2.20 (19.00)^b^					
Nimodipine^®^ tablet (30 mg NIM/1 tablet)	30.0	30.38	101.28	1.0	1.02	102.00
	40.0	39.18	97.95	2.0	1.99	99.50
	50.0	49.99	99.97	3.0	2.98	99.33
				4.0	4.02	100.50
mean		99.73				100.33
± s.d.		1.68				1.23
% RSD		1.68				1.23
% error		0.97				0.62
*t*-test	0.52 (2.571)^b^					
*F*-value	1.87 (9.55)^b^					

^a^ Mean of three determinations.

^b^The values in parentheses are the tabulated *t-* and *F*-values at *p* = 0.05.

## 4. Conclusion

In summary, spherical and hydrophilic NSCDs were successfully synthesized by hydrothermal treatment of both TSC and CA. The NSCDs exhibit high fluorescence QY (0.58), excellent aqueous solubility, excitation-dependent emission behaviour, and photostability. The prepared NSCDs can be potentially used as a promising and ideal fluorescent probe for the determination of each of NIC, NIF and NIM. The interaction of the functional groups of NSCDs with Nitro-CCBs induced quenching of the native fluorescence of the synthesized NSCDs owing to IFE (resulting from feasible overlapping between the excitation fluorescence spectrum of NSCDs and absorption bands of drugs) and static quenching effect. Subsequently, the apparent concluding features of the developed method are novelty, eco-friendliness, cost-effectiveness, simplicity and sensitivity for monitoring of Nitro-CCBs in their pharmaceutical formulations without the need for prior derivatization or use of chromogenic reagent.

## Data Availability

Data are available from the Dryad Digital Repository: https://doi.org/10.5061/dryad.pvmcvdnph [55]. The data are provided in electronic supplementary material [56].
